# Structural mass spectrometry approaches to understand multidrug efflux systems

**DOI:** 10.1042/EBC20220190

**Published:** 2023-03-29

**Authors:** Benjamin Russell Lewis, Ryan Lawrence, Dietmar Hammerschmid, Eamonn Reading

**Affiliations:** Department of Chemistry, King’s College London, Britannia House, 7 Trinity Street, London SE1 1DB, U.K.

**Keywords:** antimicrobial resistance, drug efflux, mass spectrometry, membrane proteins

## Abstract

Multidrug efflux pumps are ubiquitous across both eukaryotes and prokaryotes, and have major implications in antimicrobial and multidrug resistance. They reside within cellular membranes and have proven difficult to study owing to their hydrophobic character and relationship with their compositionally complex lipid environment. Advances in structural mass spectrometry (MS) techniques have made it possible to study these systems to elucidate critical information on their structure–function relationships. For example, MS techniques can report on protein structural dynamics, stoichiometry, connectivity, solvent accessibility, and binding interactions with ligands, lipids, and other proteins. This information proving powerful when used in conjunction with complementary structural biology methods and molecular dynamics (MD) simulations. In the present review, aimed at those not experts in MS techniques, we report on the current uses of MS in studying multidrug efflux systems, practical considerations to consider, and the future direction of the field. In the first section, we highlight the importance of studying multidrug efflux proteins, and introduce a range of different MS techniques and explain what information they yield. In the second section, we review recent studies that have utilised MS techniques to study and characterise a range of different multidrug efflux systems.

## Introduction

Membrane proteins are part of, or interact with, complex cellular membranes and account for approximately 30% of human proteins [1]. They are essential for diverse cellular functionalities and embody in excess of 60% of all drug targets [2]. Efflux pumps are membrane proteins that mediate the internal regulation of their cellular environments, and their biological importance is corroborated by their presence across all three domains of life [3]. These polytopic proteins play an indispensable role in the maintenance of cellular integrity through the transport of noxious substances to the external environment. These pumps can be specific for a singular substrate or identify and discharge a wide range of structurally unrelated drugs and, therefore, can be associated with multidrug resistance (MDR) [4].

**Figure 1:**
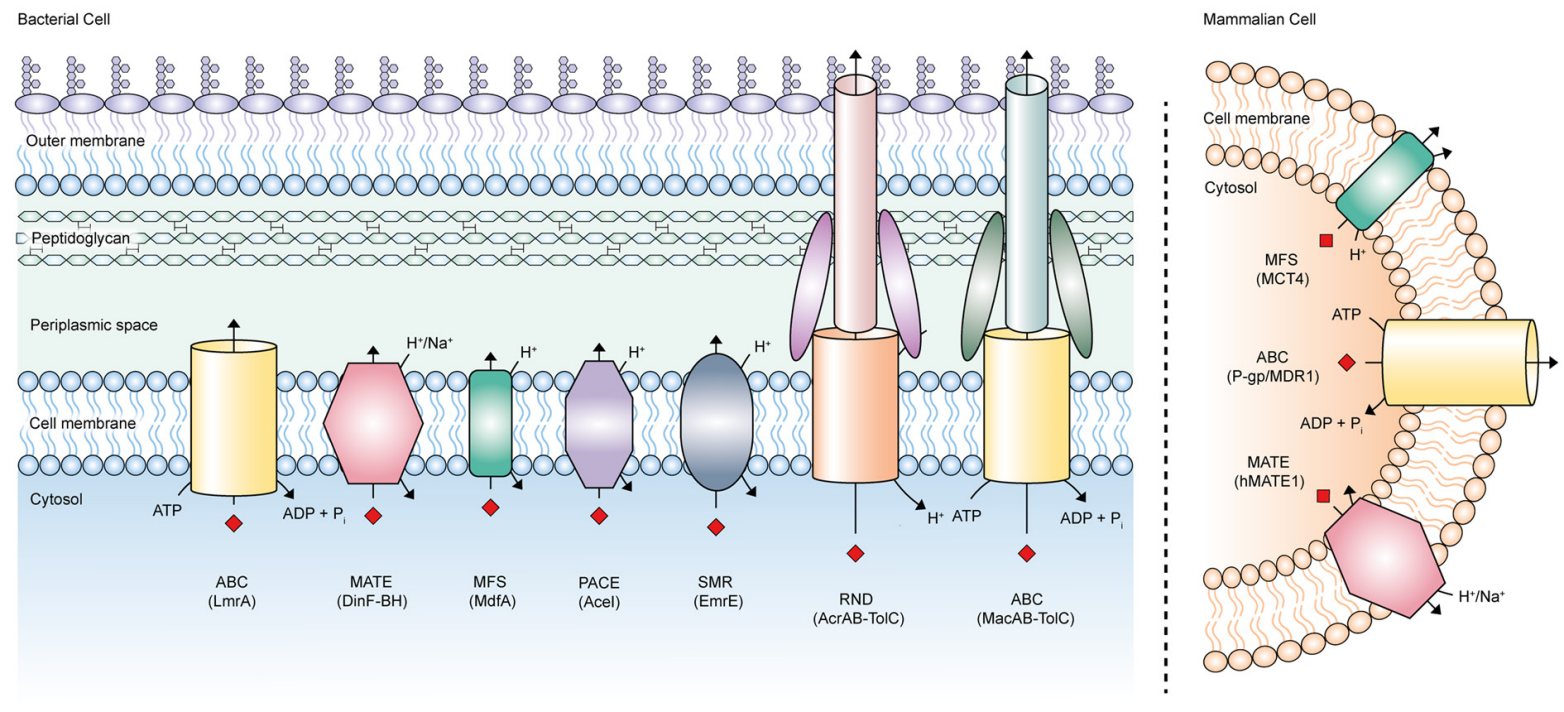
Families of MDR efflux pumps This schematic delineates the efflux pumps found within the membranes of both bacterial and eukaryotic cells that are responsible for the acquisition of MDR, along with their associated energy-coupling mechanisms. Examples of each pump are detailed in brackets. The MFS family of transporters is demonstrated as a human symporter facilitating substrate export as observed in cancer cells [88]; however, this family can act as an export or import transporter functioning via uniport, symport, or antiport. Abbreviations: MDR, multidrug resistance; ABC, ATP-binding cassette; MATE, multidrug and toxin extrusion; MFS, major facilitator superfamily; PACE, proteobacterial antimicrobial compound efflux; RND, resistance-nodulation-cell division; SMR, small multidrug resistance.

In bacteria, the ATP-binding cassette (ABC) [5], multidrug and toxin extrusion (MATE) [6], major facilitator superfamily (MFS) [7], proteobacterial antimicrobial compound efflux (PACE) [8], resistance-nodulation-cell division (RND) [9], and small multidrug resistance (SMR) [10] transporters comprise the families of efflux pumps associated with MDR (Figure 1). Bacterial MDR efflux pumps utilise energy from the proton/sodium motive force to catalyse substrate efflux, except the ABC family that exploit energy from ATP hydrolysis [5]. The substrate promiscuity of these pumps has a clinical implication in the acquisition of antibiotic resistance and is a major contributor to MDR development, enabling bacterial cells to tolerate several types of antimicrobial agents [4]. In addition to MDR, efflux pump activity is necessary for other bacterial behaviours, including quorum sensing, sporulation, virulence, and biofilm formation [4]. The latter of which further exacerbates the issue of MDR due to the increased protection from antibiotics afforded by growth within biofilm matrices [11–13]. This significant efflux pump-mediated development of MDR is inextricably linked to human health and has since emerged as one of the biggest threats to global public health. Accordingly, the World Health Organisation reported that by 2050, almost 10 million deaths each year could be a consequence of drug-resistant diseases [14].

Aside from their presence in bacteria, MDR efflux transporters are also implicated in the pathogenesis of various human diseases. Human efflux pumps also consist of orthologues of the ABC, MATE, and MFS families of transporters found in bacterial species that mediate the cellular efflux of xenobiotic agents (Figure 1) [15–18]. These MDR efflux pumps provide a selective advantage to cancer cells exposed to chemotherapeutic agents through the active efflux of these cytotoxic drugs, lowering their intracellular concentrations to subtoxic levels and conferring drug resistance [19,20]. In addition to circumventing cancer treatments, multiresistance protein-1/2 and permeability glycoprotein (P-gp) of the ABC superfamily can also limit the intracellular accumulation of human immunodeficiency virus protease inhibitors, an essential element of antiretroviral therapy [21,22]. Collectively, these efflux pumps therefore embody markers of poor prognosis of several human diseases and are often linked to poor clinical outcomes in the responsiveness to therapeutic agents.

The activity of MDR efflux pumps therefore plays essential roles in the progression of various diseases, propelling a need for research into the acquisition of MDR by such mechanisms as efflux pump activity. The application of mass spectrometry (MS)-based structural biology techniques allows for an interrogation of the structural and dynamic intricacies of these efflux pumps [23], essential in uncovering novel targets of inhibition and the design of enhanced efflux pump inhibitors (EPIs). Although this is not without its difficulties, such targeted designs of novel inhibitors offer an effective approach to recapitulate the activities of previously inefficacious inhibitors and facilitate their efficient intracellular accumulation.

## Introduction to structural MS

**Figure 2:**
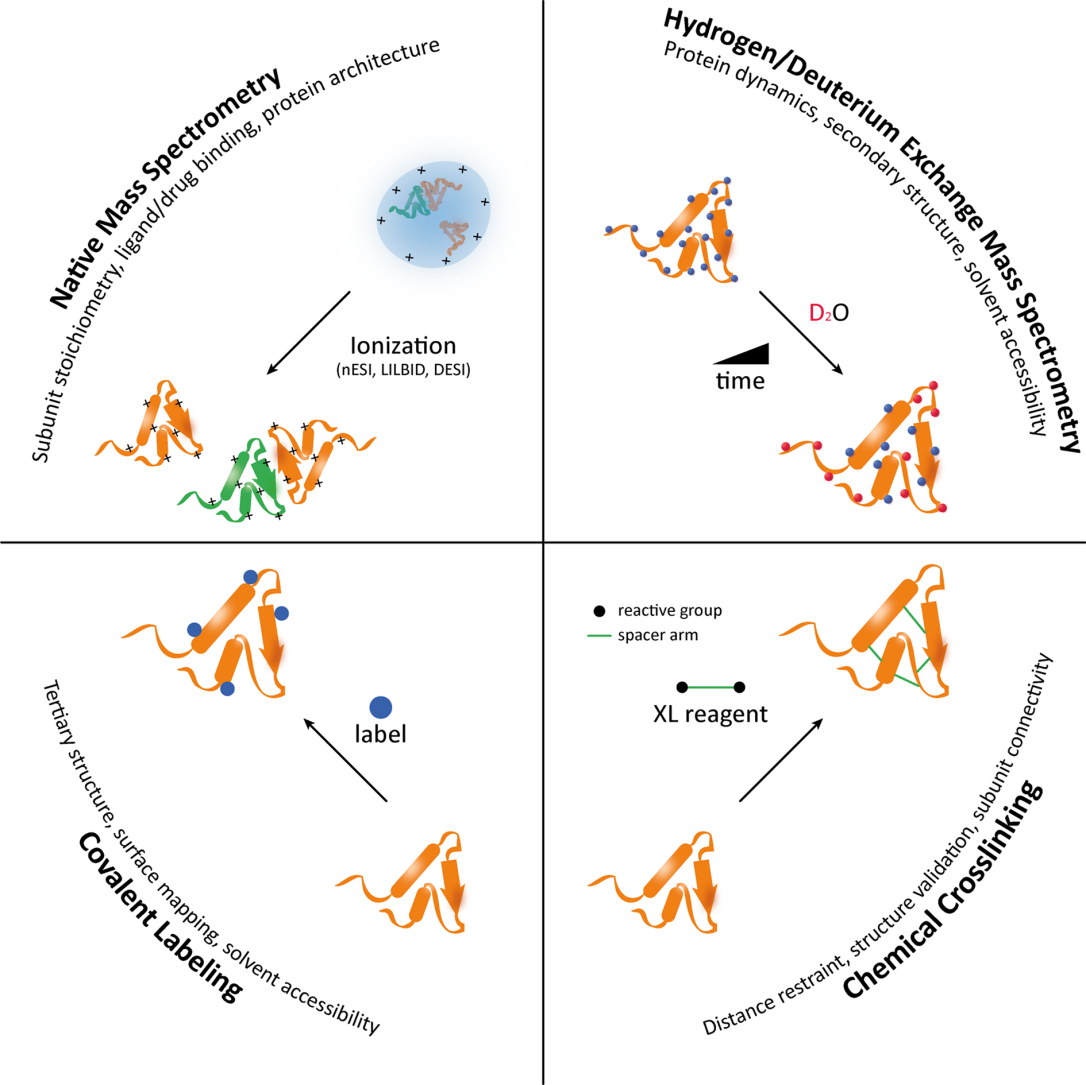
Structural MS toolbox Native MS preserves noncovalent protein interactions during the transition to the gas phase, and reports on subunit stoichiometry, ligand binding, and protein architecture. Hydrogen/deuterium exchange (HDX) MS involves the substitution of amide hydrogens on the peptide backbone for deuterium, reporting on protein structural dynamics. Chemical cross-linking (XL) MS requires the use of a chemical cross-linker to provide information on distance restraint and structure validation. Covalent labelling MS allows for surface mapping and reports on tertiary structure. Abbreviations: ESI, electrospray ionisation; nESI, nano-electrospray ionisation; LILBID, laser-induced liquid bead ion desorption; DESI, desorption electrospray ionisation; MS, mass spectrometry.

MS has emerged as a versatile tool in structural biology over the last two decades. Instrumental and methodological advancements have paved the way for new structural MS approaches that are now no longer limited to soluble proteins only, but also amenable to membrane proteins in relatively more complex lipid environments, which is of particular interest for MDR efflux pumps. The developed MS methods can generally be classified into native (non-labelling) and labelling MS (Figure 2). We provide a brief introduction into the techniques below but for a more in-depth discussion of studying membrane proteins by structural MS, the interested reader is directed to two excellent review articles [24,25].

Native MS exploits soft ionisation techniques – such as nano-electrospray ionisation (nESI) [26], laser-induced liquid bead ion desorption (LILBID) [27], and desorption electrospray ionisation (DESI) [28] – which are capable of preserving the solution-phase protein structure during the transfer into the gas phase [29]. This makes native MS an ideal method to study: (i) subunit stoichiometry [30] and (ii) protein-ligand or -drug binding [31]. Coupling native MS with ion mobility provides additional low-resolution information on the overall protein architecture [32] and even stability, when combined with collisional activation [33].

Labelling MS provides structural information based on an introduced protein label that is typically detected on the peptide level to increase spatial resolution. Essentially, three techniques can be distinguished, namely HDX, chemical XL, and covalent labelling. In HDX, a protein is diluted into a deuterated buffer enabling H/D exchange of labile backbone amides that strongly depends on a proteins’ secondary structure and solvent accessibility. Subsequent analysis requires sample quenching to acidic pH and cold temperatures (typically pH 2.5 and 0°C), to minimise D- to H- back-exchange in protic solvents as the exchange reaction is bidirectional [34]. In XL-MS, a protein is cross-linked with a suitable XL reagent. All XL reagents share a common structure composed of a spacer arm flanked by two reactive groups. The defined length of the spacer imposes a distance restraint, which can then be exploited in structural modelling [35] or to validate high-resolution structures [36]. The field of covalent labelling is dominated by hydroxyl radical footprinting by laser irradiation (fast photochemical oxidation of proteins – FPOP) [37] but other labels such as carbene [38], trifluoromethyl radicals (^●^CF_3_) [39], N-ethylmaleimide [40], diethylpyrocarbonate [41] have also been established, all of which with their own amino acid site specificities for protein surface mapping.

Membrane protein characterisation by structural MS is relatively more challenging as the membrane/lipid environment requires specific adaptations to the workflow. For Native MS, ionisation into the gas-phase is required before mass detection; the membrane proteins are still surrounded by a hydrophobic vehicle during this process, e.g. detergent micelles [42], amphipols [43], nanodiscs [44], and ‘native’ nanodiscs [45], which needs to be gently stripped off by careful optimisation of instrument parameters [46]. For HDX-MS, their lipid environment has long been a hurdle as lipids may hamper protein digestion and/or chromatographic peptide separation. However, recent advances have overcome this problem by allowing phospholipids to be removed predigestion using ZrO_2_ beads [47], size-exclusion chromatography [48], or TCA precipitation [49], which will help to make membrane protein characterisation in HDX-MS more routine. XL and chemical labelling are relatively more tolerable towards membrane proteins as enough time is available for processing the permanently labelled protein for subsequent MS analysis. However, XL of membrane proteins generally require complexes with large regions outside the membrane [50] as cross-linking of membrane domains might sterically be hindered by the lipid bilayer.

## Understanding efflux function and drug interactions

MS techniques can be deployed to reveal critical biological information regarding the function and assembly of multidrug efflux systems. Recent work has utilised a mixture of different MS techniques to characterise multidrug efflux pumps, with great success. HDX-MS is an increasingly popular technique often used to monitor protein structural dynamics over time [51,52]. Mehmood et al. were the first group to use HDX-MS to investigate the dynamics of full-length bacterial multidrug ABC transporter, BmrA, with bound and unbound ATP, in detergent micelles [53]. Their HDX data supported BmrA’s transition from an inward-facing resting state (unbound) to an outward-facing active state (bound). Interestingly, their data revealed regions in two intracellular subdomains (ICD1 and ICD2) that exhibited a large degree of flexibility in the resting state – previously these regions were thought to exist in similar conformations in both states. A recent study of BmrA by Waqas et al. utilised HDX-MS to further probe conformational states during the ATPase cycle [54]. By using a combination of HDX-MS and small-angle neutron scattering, they were able to reveal the main steps of the catalytic cycle and highlight the importance of an ADP bound inward-facing conformation adopted by BmrA during the posthydrolytic step.

Aside from prokaryotic multidrug efflux proteins, there has been success in studying mammalian efflux transporters too. P-gp was the first identified mammalian ABC transporter, and it has been linked to a variety of pathologies such as tumour multidrug resistance [20,55]. Kopcho et al. used HDX-MS to monitor the structural dynamics of P-gp in three distinct conformational states [56]. Similar to the ABC transporter-mentioned above, P-gp was determined to exhibit inward-facing, prehydrolytic, and outward-facing conformations. HDX-MS studies achieved very high coverage of P-gp in these three states, providing a comprehensive view of structural dynamics across their catalytic cycle. The starkest thing they observed was with P-gp’s extracellular loops (ECL): compared with the apo, inward-facing state, the ECLs have decreased dynamics in the prehydrolytic state but increased dynamics in the outward-facing state. The HDX-MS results implied that P-gp is occluded from the extracellular environment and the intracellular environment in the prehydrolytic state; this indicated a mechanism that prevents P-gp acting as a transporter in the intermediate transition state.

**Figure 3:**
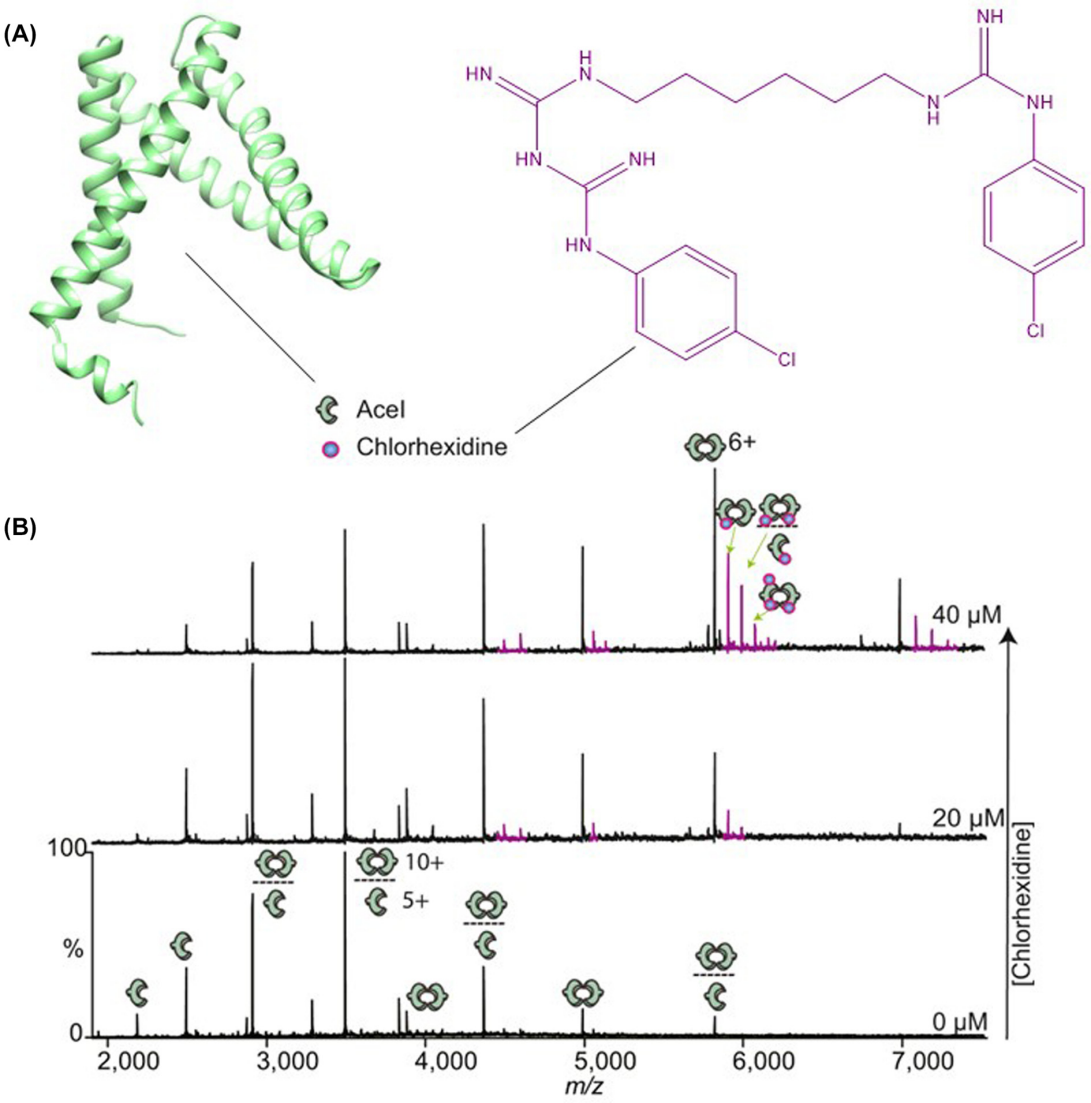
Chlorhexidine binding to AceI monitored by native MS (**A**) Structures of AceI as predicted by AlphaFold [89,90] and chlorhexidine. (**B**) Mass spectra of 5 μM AceI with increasing amounts of chlorhexidine, from 0 to 40 μM. AceI presents as a mixture of monomers and dimers. Satellite peaks next to the main charge state distribution of AceI correspond to the mass of chlorhexidine, and hence represent AceI-chlorhexidine complexes. Chlorhexdine increases the proportion of AceI dimers in the mass spectrum. Adapted with permission from Bolla et al. [57].

Aside from HDX-MS, other MS techniques have had success in studying efflux proteins. Native MS can be used to monitor protein complexes and interactions with substrates. A recent study by Bolla et al. used native MS to study the PACE protein, AceI, and its binding to nucleic acids, lipids, and drugs, under a range of conditions [57]. They found that AceI exists as an equilibrium of monomers and dimers, that it is modulated by pH (between pH 5–9), the presence of cardiolipin and binding of its chlorhexidine substrate (Figure 3). Furthermore, they showed that other cationic biocide compounds could not bind and looked at *aceI* transcription regulation by AceR. Native MS results showed AceR binds to DNA, which disrupts RNA polymerase interacting with the promoter. However, in the presence of chlorhexidine, AceR tetramerises that effects DNA binding, thus allowing the RNA polymerase to bind the *aceI* promoter again.

**Figure 4:**
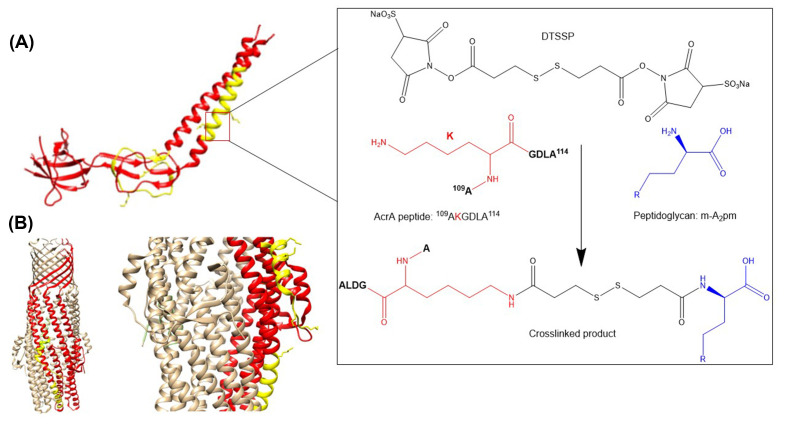
Mapping the binding sites of AcrA and TolC to PG with XL-LC/MS-MS (**A**) The crystal structure of AcrA (PDB: 2F1M), with the yellow areas representing peptides that interact with PG. Insert displays how DTSSP reacts with primary amines to create the cross-linked product. Lysine residues which can react with DTSSP have their side chains shown. (**B**) The crystal structure of TolC (PDB: 1EK9), with the yellow areas representing peptides that interact with PG. Only one protomer in the TolC trimer is coloured for clarity, and the mapped peptides can be seen more clearly in the zoomed in insert. Adapted from Shi et al. with permission [91]. Abbreviations: XL, cross-linking; LC/MS-MS, liquid chromatography tandem mass spectrometry.

XL-MS is often used to reveal sites of interactions between substrates and proteins. In 2019, Shi et al. used *in vivo* cross-linking of AcrA and TolC proteins, components of the AcrAB-TolC efflux pump (along with AcrB) from the RND superfamily [58], to peptidoglycan (PG) with bifunctional 3,3-dithiobis(sulfosuccinimidyl proprionate) (DTSSP), and analysed the interactions through protein digestion and analysis of peptide–PG cross-links by liquid chromatography tandem mass spectrometry (LC/MS-MS) [59]. DTSSP reacts with primary amines in lysine residues of proteins and the peptide linker in PG (Figure 4). They were able to confirm AcrA and TolC interactions with PG, which agreed with their single particle cryoelectron microscopy structures, and they were able to map the proteins binding sites with PG.

Aside from elucidating biological information regarding efflux pump function, MS techniques can be useful in inhibition mechanism studies. EPIs aim to inhibit multidrug efflux systems to ‘revive’ the activities of various pre-existing antibiotics that exhibit resistance [60]. This is a very promising area of research in the fight against antibiotic resistance. A recent study by Reading et al. utilised both native MS and HDX-MS to further understand AcrB inhibition by phenyl-arginine-β-naphthylamide (PAβN) [23]. Their HDX-MS data supported an inhibitory mode of action by which PAβN restricts AcrB structural dynamics, particularly in the drug-binding pockets and connecting/switch loops. Furthermore, HDX-MS studies of AcrB with both ciprofloxacin antibiotic and PAβN showed ciprofloxacin did not affect inhibition by PAβN. These HDX-MS data were further supported by fluorescence binding, molecular dynamics (MD) simulations and docking studies; this suggested the presence of a protein–EPI–antibiotic complex. This endorsed the theory RND pump inhibitors act through ‘altered dynamics’ rather than competitive inhibition and exemplified the power of HDX-MS when used in conjunction with other techniques.

## Investigating the relationship between efflux pumps and their membrane

As membrane proteins, it is important to explore the relationship multidrug efflux systems have with their cell membranes. Lipids are essential for the function of membrane proteins; the phase/presence of particular phospholipids has an effect, as does the thickness of the surrounding bilayer [61]. For efflux pumps specifically, it has been shown that lipid–protein interactions can affect their proton-dependent and substrate-dependent conformational dynamics [62]. Advances in MS techniques have made it possible to study efflux pump proteins and their interactions with lipids.

The Robinson group pioneered MS for the characterisation of lipid interactions with intact membrane protein complexes purified in detergent micelles [63–66]. Their approaches have been adopted by many MS practitioners to investigate lipid and drug interactions with membrane proteins. Using these methods, they were able to study mammalian P-gp drug and lipid binding [67]. Here, they utilised ion mobility MS to determine how size affects lipid binding to P-gp. Their results showed that small lipids (with the same net charge) bound more favourably, indicating that steric effects are important. As mentioned previously, MS techniques are very powerful when used in conjunction with other structural data. Debruycker et al. determined the crystal structure of a prototypical bacterial multidrug transporter of the MFS family, LmrP and discovered the binding cavity contained an embedded lipid [68]. They deployed native MS to investigate the presence and nature of the bound lipid, and their data revealed a phosphatidylglycerol molecule bound with high affinity to the protein interior. A recent study by Lyu et al. in 2022 used native MS to determine the binding affinities between the ABC transporter MsbA and a range of lipids found in *E. coli*, as the selectivity of lipid binding is poorly understood. They reveal that MsbA copurifies with copper(II) and MsbA has one high affinity copper(II)-binding site per subunit. Furthermore, they observed enhanced binding affinity to lipids to fully copper(II)-loaded MsbA that, combined with an X-ray structure of MsbA, revealed conformation-dependent selectivity of lipid binding, particularly for a precursor in LPS biogenesis.

**Figure 5:**
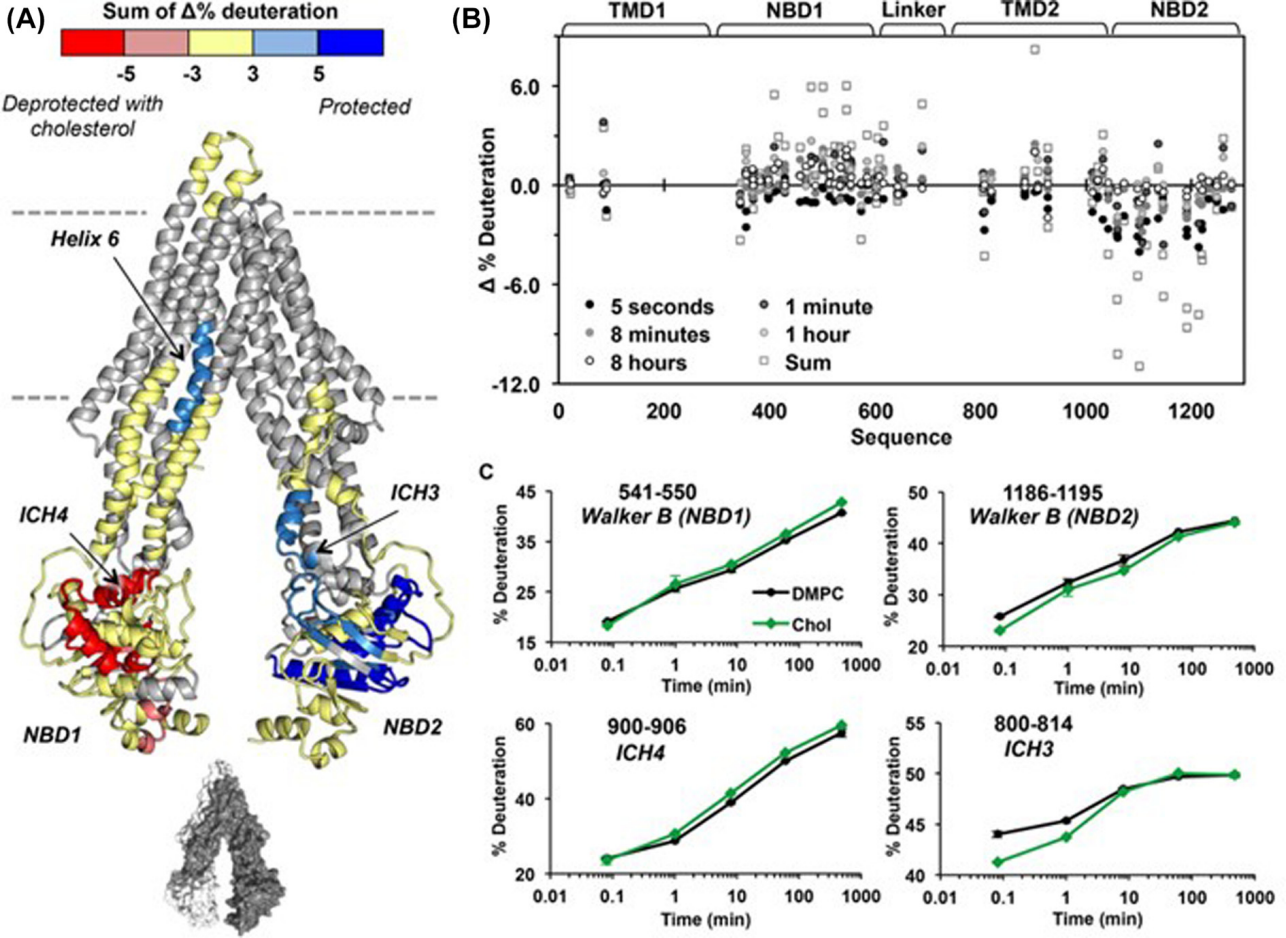
The effect of cholesterol on the structural dynamics of P-gp (**A**) Structure of P-gp (PDB: 5KPI) with the differential hydrogen deuterium exchange (ΔHDX) painted on. ΔHDX = P-gp cholesterol nanodiscs – P-gp DMPC only nanodiscs. Deprotected peptides are coloured red, protected peptides are coloured blue, areas with insignificant ΔHDX are coloured yellow, and regions not covered are grey. Only peptides with significance across two or more timepoints were deemed ‘affected’ (95% significance). The greyscale structure at the bottom represents the two halves of P-gp: white = residues 1–650 and grey = residues 651–1284. (**B**) Woods plot of the ΔHDX data across all timepoints and the sum of all timepoints. The deuterium uptake for the peptides is shown on the Y-axis and the sequence of the peptides shown on the X-axis. The domains of the corresponding peptides are labelled at the top. (**C)** Peptide uptake plots for four peptides across different domains. Peptide uptake plots show percentage deuteration as a function of time for a given peptide. Obtained with permission from Biochemistry [75].

Traditionally, the study of membrane proteins required the presence of a mimetic membrane environment, such as detergent micelles [69,70]. However, these lack the native lipid environment of the protein, which can affect folding, structure, function, and dynamics of membrane proteins [64]. An alternative mimetic is the membrane scaffold protein (MSP) nanodisc, which are self-assembled proteolipid particles, containing MSP proteins encapsulating a well-defined mixture of phospholipids [71,72]. Hebling et al. first described an experimental workflow for HDX-MS studies in MSP nanodiscs, which involves removing lipids using ZrO_2_ beads [73], this protocol being used by Li et al. to investigate P-gp structural dynamics in a lipid milieu [74]: their data provided the first look at the energy landscape of free P-gp in a lipid environment, finding a minimum of three conformational states. Furthermore, bimodal ‘EX1’ kinetics in the raw HDX-MS data was observed over a wide time scale for multiple peptides, suggesting the energy landscape is likely even more complicated. A more recent study by Clouser et al. delved deeper by investigating the effect of cholesterol in the membrane on P-gp structural dynamics in lipid nanodiscs (Figure 5) [75]. They observed long-range dynamic changes in the nucleotide binding domain of P-gp, correlating the previously seen effects of lipid composition on activity. If the reader is interested in learning more about HDX-MS kinetics; then, we recommend the excellent review by Wales and Engen [76].

Another alternative mimetic is the styrene maleic lipid particle (SMALP) native nanodiscs, which use SMA polymer to directly solubilise proteins from their membrane, in a detergent-free manner, providing nanodiscs that contain a subset of its native lipids. Reading et al. benchmarked a workflow for HDX-MS on membrane proteins in SMALPs [77] using the GlpG membrane protein, and showed that specific protein regions were sensitive to changes in its native lipid environment. As well as HDX-MS, advances in native MS have allowed the study of efflux proteins in SMALP nanodiscs. LILBID and nESI were shown to be adept at preserving intact membrane proteins as they are transferred to the gas phase, in detergent or encapsulated in either MSP nanodiscs and SMALP nanodiscs [25,45,78,79]. Henrich et al. used LILBID-MS to elucidate the oligomeric state of the EmrE efflux pump within MSP nanodisc environments, visualising mixed oligomers for EmrE (monomers and dimers), and the AcrB efflux pump, confirming its trimeric structure with surrounding lipids attached. These studies revealed that proteins in SMALPs require larger energy to be released from their environment compared with MSP nanodiscs and detergent micelles, likely due to the increased stability of a protein being surrounded by its native environment, highlighting the importance of this in the study of efflux proteins. For a comprehensive review of polymer (‘native’) nanodiscs, we recommend the excellent review by Dimitrova et al. [80].

## Future outlooks

**Figure 6:**
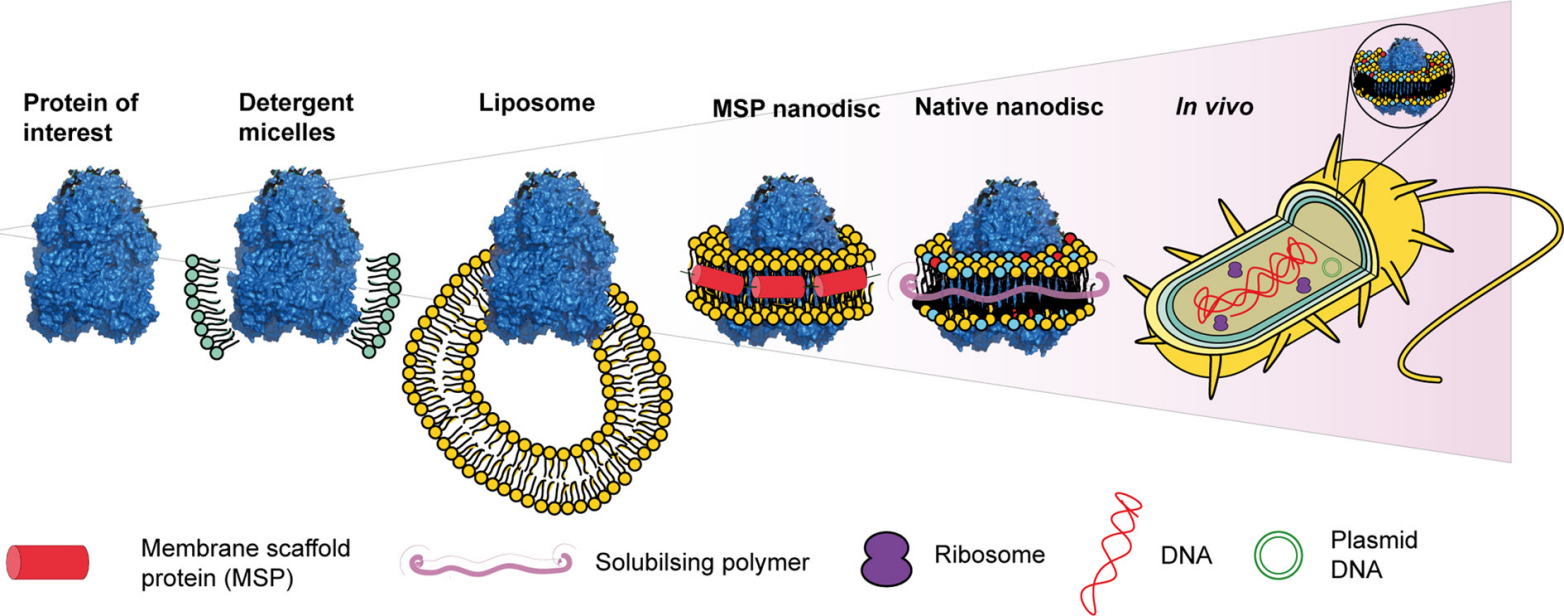
The increasing complexity of studying efflux protein systems Schematic showing the different environments used to study membrane proteins, with increasing complexity and representability.

MS is becoming increasingly important to a structural biologist’s toolkit, with a multitude of techniques available to elucidate different sorts of information on multidrug efflux systems. MS techniques are strongest when used in conjunction with complementary techniques, such as MD simulations, cryo-EM, X-ray crystallography, and/or functional assays. It is now possible to study efflux pumps in a range of different *in vitro* environments by using a variety of techniques. However, the biggest challenge is the aim to study multidrug efflux systems *in situ* (Figure 6). Isolating proteins from their native environment into membrane mimetic systems may vary their functions, and is not representative of their state *in vivo* [81]. There have been recent advances in the applications of MS based methods in the study of membrane proteins *in situ*.

Work by Chorev et al. developed an approach to eject protein assemblies from their native environments for analysis by MS [82]. This approach utilises membrane protein-enriched extracellular vesicles and has already been applied to mammalian cells and both inner and outer *E. coli* membranes. They were able to uncover subassemblies of both the AcrAB-TolC and MdtAB-TolC multidrug efflux pumps. The fact both complexes stay partly intact in the gas phase provides a promising new approach to monitor the impact of EPIs on the assembly of these systems. Moreover, Native-liquid extraction surface analysis MS imaging workflows can be used to provide spatial, conformational, and mass information on proteins directly from thin tissue sections [83]. While it has not been applied to efflux proteins yet, this provides another option for future study. Furthermore, this technique has been adapted nano-DESI for increased resolution [84].

There has also been progress in HDX-MS towards an increasingly complex, ‘in cell’ approach. The first example was in 2001, where Ghaemmaghami et al. studied the thermodynamic stability of model protein λ_6_-_85_ *in vitro* and in the *E. coli* cytosol [85]. They found little difference between *in vitro* and in cell, but when the *E. coli* were placed in a hyperosmotic environment, the thermodynamic stability of λ_6_-_85_ was greatly enhanced. Donnarumma et al. were able to characterise outer membrane protein OmpF, in outer membrane vesicles naturally released by *E. coli*. By analysing the deuterium uptake of OmpF in its native environment, they were able to report areas of the protein that were buried or exposed, which was in good agreement with previous X-ray diffraction data. Recent work by the Sosnick group has studied the TonB-dependant transporter, BtuB, using HDX-MS with increasing complexity [86,87]. One method was measuring BtuB structural dynamics within the *E. coli* outer membrane, similar to the study by Donnarumma et al. The second method used a protocol developed for *in vivo* HDX-MS. By over expressing cells with BtuB and diluting into deuterated LB buffer, they achieved desired deuterium labelling. They proposed that BtuB binding to B12 was able to break a salt bridge between Arg14 and Asp316 in both methods, whereas in reconstituted systems B12 does not bind.

Taken together, these studies highlight the importance to strive for native environments and support that developing methods that can achieve *in situ* information is likely to be highly valuable for studying efflux systems.

## Summary

- Multidrug efflux pumps are ubiquitous across prokaryotes and eukaryotes and have major implications in diseases such as multidrug-resistant infections and tumours.
- There are a variety of MS techniques available to elucidate critical biological information about these systems.
- Efflux pump systems can be studied in a variety of different *in vitro* membrane mimetic environments, ranging from detergent solubilised to (native) nanodiscs.
- There is a push towards monitoring these systems within their cellular contexts using mass spectrometry.

## Abbreviations

ABC: ATP-binding cassette
DESI: desorption electrospray ionisation
DTSSP: 3,3-dithiobis(sulfosuccinimidyl proprionate)
ECL: extracellular loop
EPI: efflux pump inhibitor
FPOP: fast photochemical oxidation of proteins
HDX: hydrogen deuterium exchange
LC/MS-MS: liquid chromatography tandem mass spectrometry
LILBID: laser induced liquid bead ion desorption
MATE: multidrug and toxin extrusion
MDR: multidrug resistant
MFS: major facilitator superfamily
MS: mass spectrometry
MSP: membrane scaffold protein
nESI: nano-electrospray ionisation
P-gp: P-glycoprotein
PACE: proteobacterial antimicrobial compound efflux
PAβN: phenyl-arginine-β-naphthylamide
PG: peptidoglycan
RND: resistance nodulation and cell division
SMALP: styrene maleic acid lipid particle
SMR: small multidrug resistance
XL: cross-linking
